# Ethnomedicinal Plants with Protective Effects against Beta-Amyloid Peptide (Aβ)1-42 Indicate Therapeutic Potential in a New In Vivo Model of Alzheimer’s Disease

**DOI:** 10.3390/antiox11101865

**Published:** 2022-09-21

**Authors:** Norah A. Althobaiti, Farid Menaa, Johnathan J. Dalzell, Aishah E. Albalawi, Hammad Ismail, Mousa A. Alghuthaymi, Reem D. Aldawsari, Haroon Iqbal, Claire McAlinney, Brian D. Green

**Affiliations:** 1 Institute for Global Food Security, School of Biological Sciences, Queen’s University Belfast, Biological Sciences Building, Chlorine Gardens, Belfast BT9 5DL, UK; 2 Biology Department, College of Science and Humanities-Al Quwaiiyah, Shaqra University, Al Quwaiiyah 19257, Saudi Arabia; 3 Departments of Internal Medicine and Advanced Technologies, Fluorotronics-California Innovations Corporation, La Jolla, San Diego, CA 92037, USA; 4 Department of Grassland and Plant Science, AgriFood and Biosciences Institute, Belfast BT9 5PX, UK; 5 Biology Department, Faculty of Science, University of Tabuk, Tabuk 71491, Saudi Arabia; 6 Department of Biochemistry and Biotechnology, University of Gujrat, Gujrat 50700, Pakistan

**Keywords:** *G. pallida*, Alzheimer’s disease, phytotherapy, ethnomedicine, amyloid-beta peptide, oxidative stress, chemosensing, survival, antioxidant response

## Abstract

Alzheimer’s disease (AD) is a progressive neurodegenerative disorder with unmet medical need. This investigation consisted of testing a range of ethanolic ethnomedicinal plant extracts (*n* = 18) traditionally used in the treatment of disorders such as anxiety, delirium, and memory loss. They were then screened for in vitro inhibitory activity against acetylcholinesterase (AChE), butylcholinesterase (BuChE), beta-secretase 1/beta-site amyloid precursor protein (APP) cleaving enzyme 1 (BACE1), and antioxidant activities. Plants with potent activities were further characterised using a recently developed in vivo model of AD, *Globodera pallida*. The ability of phytoextracts to protect this organism against amyloid-beta Aβ (1-42) exposure was assessed by measuring chemosensing, survival rate, production of reactive oxygen species (ROS), and antioxidant responses. Extracts (*n* = 5) from *Juglans regia* (leaves), *Ellettaria cardamomum* (seeds), *Cinnamomum zeylanicum* (bark), *Salvia officinalis* (leaves/flowers), and *Hypericum perforatum* (flowers) exerted concentration-dependent inhibitory activities against AChE and BuChE. Three of these plant extracts (i.e., *J. regia*, *E. cardamomum*, and *S. officinalis*) possessed strong concentration-dependent inhibitory activity against BACE1. Furthermore, the five selected medicinal plant extracts not only enhanced significantly (*p* < 0.05) the nematode’s chemosensing, survival rate, and antioxidant responses (i.e., anti-ROS production, mitochondrial reductase activity, oxidized glutathione (GSSG) to reduced glutathione (GSH) ratio), but also greatly restored (*p* < 0.05) in a concentration-dependent manner the Aβ (1-42)-induced deleterious changes in these same parameters. In brief, this investigation highlights plant extracts with strong anti-AD activities which could be trialled as novel therapeutic supplements or undergo further biodiscovery research.

## 1. Introduction

Therapeutical options for Alzheimer’s disease (AD) are currently quite limited. The current available medications (e.g., AchE inhibitors such as galantamine, rivastigmine, and donepezil) often provide symptomatic relief, and some may slow disease progression, but long-term use can be associated with side effects and diminished effectiveness. The development of novel, safe, and cost-effective therapies for AD is a global public health priority [1]. Natural products represent potential sources of novel lead compounds, and there is a track record of these being developed into clinically used drugs. For instance, certain medicinal plants have proven to be useful sources of acetylcholinesterase (AchE) inhibitors such as galantamine [2,3,4,5].

The considerable unmet medical needs in AD have increased scientific and public interest in alternative remedies such as nutraceutical supplements and herbal remedies. There are examples of products marketed as ‘memory enhancers’ or as treatments for delaying or preventing dementia. Over-the-counter herbal and nutraceutical supplements such as ginseng, ginkgo biloba, oils rich in omega 3 fatty acids, Huperzine A, amino acids, vitamins, and choline are purported to maintain brain health and/or delay dementia [6]. Medical foods are a category of products prepared from GRAS (generally recognised as safe) substances, which are intended for the specific dietary management of a disease or condition that has distinctive nutritional requirements. In US and European markets, Axona (Accera, Inc, Vevey, Switzerland), Souvenaid (Danone Research, Paris, France), and CerefolinNAC (Covington, LA, USA) represent specific products which are marketed for AD.

In some countries, up to 80% of the population depends on herbal remedies as a primary form of healthcare [7]. Traditional medicines have been used for thousands of years in the treatment and prevention of human diseases, including AD [8]. There is a scientific underpinning here, given that several chemical compounds isolated from medicinal plants have been established to have neuroprotective actions in AD, and their mechanisms of action have been investigated [9].

The present study examined 18 medicinal plants with a history of ethnomedicinal use in the treatment of disorders such as anxiety, delirium, and memory loss. The tested plant extracts included: *Anemarrhena asphodeloides* Bunge. (rhizome), *Capsella bursa-pastoris* L. (herb), *Centella asiatica* L. (leaf), *Cinnamomum zeylanicum* L. (bark), *Curcuma* *longa* L. (rhizome), *Ellettaria cardamomum* L. (seed), *Euphorbia hirta* L. (leaf), *Euphoria longan* Lam./*Dimocarpus longan* Lour. (fruit), *Fritillaria thunbergii* Miq. (bulb), *Hypericum perforatum* L. (flower), *Hyssopus officinalis* L. (flower/leaf), *Juglans regia* L. (leaf), *Leonurus cardiaca* L. (aerial), *Lilium brownie* F.E.Br. (flower/leaf), *Paullinia cupana* L. (flower), *Salvia officinalis* L. (flower/leaf), *Uncaria rhynchophylla* Miq. (thorn/leaf), and *Zingiber officinale* Roscoe. (rhizome).

Plants extracts were initially screened for activities relevant to AD therapy. These included measuring their AchE, BuChE, and BACE-1 inhibitory activities as well as their chemosensing, survival, and antioxidant capacities. The rationale of this study was that phytoextracts exerting a range of potent multi-modal activities may be more effective than individual therapies. There are also potential downstream effects to be explored; for instance, the inhibition of AChE may protect against the damaging effects of Aβ aggregation and its neurotoxicity [10]. The most promising extracts (*n* = 9) were then shortlisted for testing in *G. pallida* as a novel and suitable non-transgenic model of AD, which is proposed to be a less complicated alternative to transgenic *C. elegans* models, and to understand the biological mechanisms underlying Aβ pathology [11,12]. This plant parasitic nematode was shown to easily assimilate exogenous amyloid peptides and mimic AD through retrograde transport along the chemosensory amphidal neurons, resulting in impaired behavioural responses to a well-established chemoattractant [11,12]. The amyloid hypothesis postulates that the neuropathology associated with AD including cognitive decline, dysregulation of energy metabolism, mitochondrial dysfunction, and neuronal cell death correlate with the extracellular deposition of amyloid-β (Aβ) and its associated neurotoxicity [13]. Aβ (1-42) is the most toxic and shows the greatest propensity to aggregate [14]. Unsurprisingly, in *G. pallida*, Aβ (1-42) had the most detrimental effects on chemotaxis, cell viability, survival rate, ROS production, and glutathione levels compared with all other Aβ isoforms [11]. The present study assessed the ability of selected medicinal plant extracts to mitigate the Aβ (1-42)-induced damage in *G. pallida* in vivo. Aβ plaques are thought to cause oxidative stress in AD neurons and increase mitochondrial damage and ROS production, which subsequently leads to neuronal cell death [15]. The *G. pallida* model also responded in this way with significantly increased levels of mitochondrial reductase activity, ROS, and glutathione (as an antioxidant defence mechanism) [11]. Therefore, the present investigation determined whether medicinal plants shortlisted from in vitro studies had therapeutic effects in *G. pallida* by examining their ability to counteract Aβ-induced impairments in chemotaxis, survival, ROS production, and antioxidant responses. The novel *G. pallida* model has been shown to respond to clinically approved therapies, but this study represents its first use in the discovery of new potential therapies.

## 2. Materials and Methods

### 2.1. Chemicals

Acetylcholinesterase (EC #: 232-559-3, AChE) from *Electrophorus electricus* (electric eel), acetylthiocholine iodide (EC #: 217-474-1, ATCI), butyrylcholinesterase from equine serum (EC #: 232-579-2, BuChE). *S*-Butyrylthiocholine chloride (EC 244-731-5: BTCC), di-nitro-thiobenzoate (EC #: 200-714-4, DTNB), galantamine (EC #: 217-780-5), and BACE1 activity detection kit (fluorescent) (product reference #: CS0010) were purchased from Sigma (Sigma–Aldrich, Dorset, UK). KF-14 (Lys-Thr-Glu-Glu-Ile-Ser-Glu-Val-Asn-Sta-Val-Ala-Glu-Phe) was custom synthesised (GL Biochem, Shanghai, China) as a positive control in assays. All solvents used were of analytical grade. Phosphate-buffered saline (1× PBS; 0.1 M) was prepared, adjusted to pH 8.0, and stored at ≤ 15 °C.

### 2.2. Plant Extracts 

All plants materials were obtained from Herbal Apothecary (Herbal Apothecary, Kent, UK). These were: *Anemarrhena asphodeloides* (rhizome), *Capsella bursa-pastoris* (herb), *Centella asiatica* (leaf), *Cinnamomum zeylanicum* (bark), *Curcuma longa* (rhizome), *Ellettaria cardamomum* (seed), *Euphoria hirta* (leaf), *Euphoria longan* (fruit), *Fritillaria thunbergii* (bulb), *Hypericum perforatum* (flower), *Hyssopus officinalis* (flower/leaf), *Juglans regia* (leaf), *Leonurus cardiaca* (aerial), *Lilium brownii* (flower/leaf), *Paullinia cupana* (flower), *Salvia officinalis* (flower/leaf), *Uncaria ryhnchophylla* (thorn/leaf), and *Zingiber officinale* (rhizome). Plant materials were pulverised to a fine powder using an analytical mill, then macerated in ethanol:water (70%: 30% (v/v)) (1 g per 5 mL) for 48 h at room temperature (18–22 °C). Extracts were Büchner filtered with Whatman™ grade 1 paper (GE Healthcare, Kent, UK), and concentrated using a rotary evaporator (40 °C). Samples were dried using a Modulyo Freeze Dryer (Thistle Scientific Ltd., Glasgow), and the yield of each extract was calculated (Table S1). For all experiments, a 5 mg/mL stock solution of each extract was prepared in Ultrapure H_2_O.

### 2.3. AChE and BuChE Inhibitory Activities

AChE and BuChE inhibitory activities were measured by the Ellman method [16]. Briefly, 125 µL of the chromogen reagent 5,5′dithiobis nitro benzoic acid (3 mM DTNB) was added to wells of a 96-well plate, followed by either 25 µL acetylthiocholine iodide substrate (1.5 mM ATCI) or 25 µL s-butyrylthiocholine chloride substrate (15 mM BTCC). Next, 50 µL phytoextracts (*n* = 18; 5 mg/mL) were diluted in 1× PBS to concentrations of 25–500 µg/mL. The diluted phytoextracts were subsequently filtered using a 0.22 µm filter and placed in the prepared 96-well plate. Enzymatic reactions were commenced by the addition of 25 µL at the optimal unit amount of either AChE (0.4 U) or BuChE (0.04 U). Plates were then placed in a plate incubator (VWR^®^ symphony^™^, Lutterworth, Leicestershire, UK) at 37 °C for 30 min. The spectrophotometric absorbance was recorded at 405 nm using a microplate reader (Tecan Safire 2, Thermo Fisher Scientific, Enderby, Leicester, UK), and the inhibitory activities were expressed as percentages.

Galantamine inhibits both enzymes and was used as the positive control (PC) at the pre-determined half-maximal inhibitory concentrations (IC50) for both AChE (18 µM) and BuChE (2 µM). Kinetic studies involved fixed concentrations of the inhibitor (18 µM or 2 µM) or selected phytoextracts (*n* = 5; 500 µg/mL) and variable substrate concentrations (i.e., acetylthiocholine iodide (ATCI): 0.0023–0.15 mM for AchE inhibition assay or s-butyrilthiocholine chloride (BTCC): 0.023–1.5 mM for BuChE inhibition assay). Sodium phosphate buffer (http://cshprotocols.cshlp.org/content/2006/1/pdb.rec8303, accessed on 22 August 2022) was used as the negative control (NC). The five selected phytoextracts were: *J. regia*, *E. cardamomum*, *C. zeylanicum*, *S. officinalis*, and *H. perforatum.* Km and Vmax were calculated for these screened plant extracts by Michaelis–Menten curve fitting. Their type of enzymatic inhibition was determined by analysis of the double reciprocal (Lineweaver–Burk) plot using Michaelis–Menten kinetics.

### 2.4. BACE-1 Inhibitory Activity

The BACE-1 enzyme activity of plant extracts was assayed by fluorescence detection kit (BACE-1 Activity Detection Kit, Sigma). Each plant extract (5 μL; pre-screening *n* = 18 at 500 µg/mL and screening *n* = 4 at 15.6–1000 µg/mL to determine their respective IC_50_), assay buffer (73 μL), and BACE-1 substrate (20 μL; 50 μM) were added to wells of a 96-well plate (PS-microplate, 96-well flat bottom, black, Greiner Bio-One, Leipzig, Germany). Reactions were initiated by the addition of the BACE-1 enzyme (2 μL; 0.3 U). Plates were incubated for 2 h at 37 °C using a plate incubator (Incubating Microplate Shaker, VWR^®^, Thorofare, NJ, USA). The fluorescence (Ex: 320 nm; Em: 405 nm) was measured with a microplate reader (Tecan Safire2^™^ Microplate Reader, Thermo Fisher Scientific, UK). A known peptide (Lys-Thr-Glu-Glu-Ile-Ser-Glu-Val-Asn-Sta-Val-Ala-Glu-Phe) inhibitor of BACE-1 (KF-14; 25 µM) was custom synthesised (GL Biochem, China) and used as the PC (solution of the buffer with the BACE-1 substrate and BACE-1/KF-14 enzyme mixture). A vehicle group (i.e., Buffer 1% DMSO in double distilled water (ddH_2_O) + BACE-1 substrate) was used as the NC. All reactions were carried out in duplicate. Kinetic studies involved fixed concentrations of the inhibitor (25 µM) or phytoextract (*n* = 4; 500 µg/mL), and the substrate was varied (0.156–10 mM). The four selected phytoextracts were: *S. officinalis, J. regia*, *E. cardamomum*, and *F. thunbergii*.

### 2.5. Antioxidant Activity

Antioxidant activity was measured in a 96-well plate by the commercial colorimetric acid 2,2′-azino-bis (3-éthylbenzothiazoline-6-sulphonique (ABTS) antioxidant assay kit (Sigma, UK) in accordance with the manufacturer’s instructions. Trolox [6-Hydroxy-2,5,7,8-tetramethylchroman-2-carboxylic acid], a water-soluble vitamin E analogue, served as a PC because it could inhibit the formation of the radical cation in a dose-dependent manner. Stock solutions of pre-screened phytoextracts (*n* = 7; 5 mg/mL) were diluted 1:2 with the kit assay buffer (2.5 mg/mL), and then serial diluted concentrations (4.3–140 µg/mL) were obtained. The seven selected phytoextracts were: *J. regia*, *E. cardamomum*, *C. zeylanicum*, *S. officinalis*, and *H. perforatum, F. thunbergii*, and *P. cupana*. Assay buffer was used as a NC. Reactions stood at room temperature (RT) for 5 min before 100 µL of Stop Solution was added. Thereafter, the absorbance was immediately measured (405 nm). The experiments were conducted in duplicate, and all results were interpolated from a Trolox standard curve (mM).

### 2.6. Chemotaxis Studies in G. pallida

Culture, maintenance, hatching, and testing of *G. pallida* have been previously described [11]. Briefly, freshly hatched second-stage juvenile (J2s) nematodes were used in each assay. For chemosensory assays, about 100 J2s were washed in spring water, centrifuged (2500 rpm; 2 min), re-suspended in spring water, and transferred to 24-well plates. Aβ (1-42) (GL Biochem, Shanghai, China) was prepared at 100 µM as well as plant extracts diluted to the concentrations indicated in the diagrams with 1% DMSO in ddH_2_O, which was also used as vehicle control. *G. pallida* J2s were exposed to Aβ (1-42) (100 µM) and/or the selected plant extracts (*n* = 5; 10–100 µg/mL) in 24-well plates at 16 °C for 24 h. The five selected phytoextracts were: *J. regia*, *E. cardamomum*, *C. zeylanicum*, *S. officinalis*, and *H. perforatum*. Petri dish (3 cm) ‘arenas’ were prepared on agar, with either water or mannitol (chemotractant), as previously described [11]. After washings, centrifugation (2500 rpm; 2 min), and resuspension in spring water, the treated *G. pallida* were transferred from the 24-well plates to the centre of the petri dish ‘arenas’, which were then left for 2 h at RT on a flat surface. Organisms on each side of the ‘arena’ were counted, and chemotaxis index (CI) was calculated as previously described [11].

### 2.7. Phytoprotective Effects against Aβ (1-42)-Induced Impairments in G. pallida

The survival rate (%) was determined by incubating, for 24 h at 16 °C, *G. pallida* J2s (50/well) in 24-well plates with 100 µM Aβ (1-42) at and/or 100 μg/mL plant extracts. The viability was estimated by naked-eye observation under a light microscope, according to a previously detailed method [11]. Live organisms had characteristic ‘body bends’ or any movement, whereas dead organisms had characteristic ‘poker straight’ shapes [11]. 

The ROS production was determined, as recently described [11], using dichloro-dihydro-fluoresceine-diacetate (DCFHDA) assay (Sigma–Aldrich, UK). Briefly, *G. pallida* J2s (100/well) were placed in black 96-well microtiter plates containing 100 µL of spring water (NC—untreated cells) or Aβ (1-42) at 100 µM and/or plant extracts at 100 μg/mL, and 50 µM DCFDA. After incubation at 16 °C, the fluorescence (Ex: 495 nm; Em: 529 nm) was measured every 30 min for 24 h, and total ROS production was analysed by calculating the area-under-the-curve (AUC).

The mitochondrial reductase activity was determined, as previously detailed [11], using Alamar Blue (Life Technologies, Paisley, UK), in accordance with the manufacturer’s instructions. Briefly, *G. pallida* J2s (100/well) were placed in 96-well plates containing 100 µM Aβ (1-42) and/or 100 μg/mL plant extracts. Alamar Blue (10 µL) was then added to each well, and the fluorescence (Ex: 530 nm; Em: 590 nm) was measured every 30 min for 24 h at 18 °C. Controls included a NC (medium control without cells—background absorbance), a vehicle control (untreated cells, which is shown in the diagrams), and a PC (100% reduced Alamar Blue).

The quantitative measurement of the total glutathione content within a sample (GSH/GSSG) was determined, as previously reported [11], using OxiSelect^™^ Total Glutathione Assay Kit (Cell Biolabs, Inc., San Diego, CA, USA), in accordance with the manufacturer’s instructions. Glutathione Reductase reduces oxidized glutathione (GSSG) to reduced glutathione (GSH) in the presence of nicotinamide adenine dinucleotide phosphate (NADPH). Subsequently, the chromogen reacts with the thiol group of GSH to produce a coloured compound that spectrophotometrically absorbs at 405 nm. The total glutathione content in unknown samples is determined by comparison with the pre-determined glutathione standard curve. Briefly, 25 µL of the 1× Glutathione Reductase solution was added to each well to be tested in 96-well microtiter plates prior to the addition of 25 µL of the 1× NADPH solution to each well to be tested. Then, 100 µL of the prepared glutathione standards or samples to be tested in *G. pallida* J2s (1000/well) (i.e., 100 µM Aβ (1-42) and/or 100 µg/mL plant extracts) was added to each well and mixed thoroughly. Untreated cells were used as a vehicle control. Eventually, 50 µL of the 1× Chromogen was added and mixed quickly. The optical density (OD)/absorbance was immediately recorded at 1–2 min intervals for 10 min. The rate of chromophore production is proportional to the concentration of glutathione within the sample. The average of each standard, sample, and background absorbance value against incubation time was graphed. The total glutathione content was calculated by determining the slopes for each value from the linear portion of each curve, subtraction of the background, then plotting the net slopes of the GSSG standards against the micromolar concentration of GSSG. Unknown concentrations were determined from the interpolation of the standard curve.

### 2.8. Statistical Analyses

All experiments were performed in triplicate. All data are expressed as mean ± standard error mean (SEM). All statistical analyses including linear/non-linear regression, enzyme kinetics (Km and Vmax), and one-way ANOVA (with Tukey’s post hoc test) were undertaken with Graphpad Prism (GraphPad, California, USA). Statistical significance and insignificance are indicated in figures: *ns* (not significant) means ^#^ *p* > 0.05, whereas * *p* < 0.05, ** *p* < 0.01, and *** *p* < 0.001 represent significant differences between groups.

## 3. Results

### 3.1. Cholinesterase Inhibitory Activities

**Figure 1:**
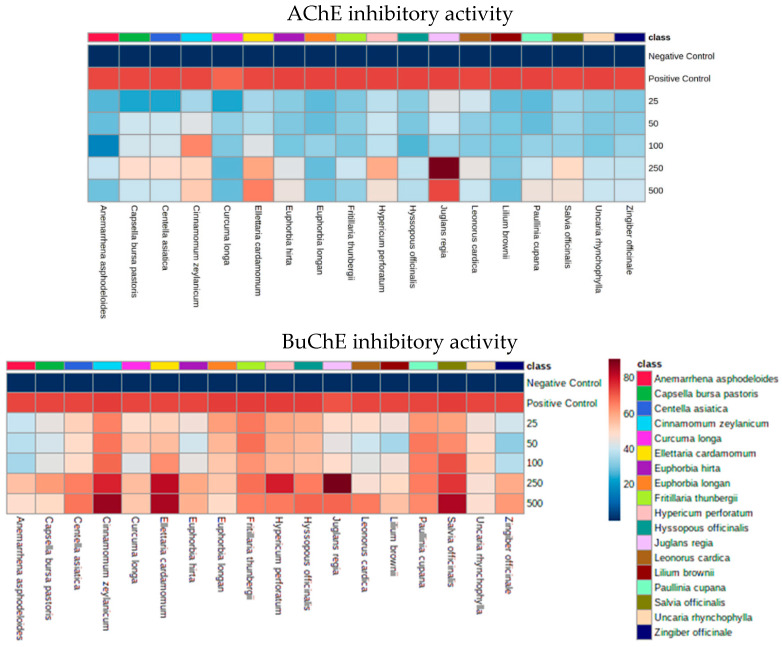
Concentration-dependent effects of plant extracts on acetylcholinesterase (AChE) and butyrylcholinesterase (BuChE) and inhibitory activities. A total of 18 plant extracts were screened using both AChE and BuChE inhibitory assays. Di-nitro-thiobenzoate was added to wells of a 96-well plate, followed by either acetylthiocholine iodide (ATCI) or butyrylthiocholine chloride (BTCC) as substrates. Phytoextracts were tested in duplicate at concentrations ranging from 25–500 µg/mL. Mean percentage inhibition data is summarised in the heatmaps. Positive control: galantamine (18 µM in AChE experiments or 2 µM in BuChE experiments); negative control: sodium phosphate buffer.

Plants extracts (*n* = 18, 25–500 µg/mL) were screened for AChE and BuChE inhibitory activities in a concentration-dependent manner (Figure 1; Tables S2 and S3). All plants extracts exhibited some degree of inhibitory activity towards both AChE and BuChE, and plant extracts with the most potent activity (*n* = 5) were *J. regia, E. cardamomum, C. zeylanicum, S. officinalis,* and *H. perforatum*. Their respective IC_50_ values for AchE and BuChE inhibitory activities were: 0.246 mg/mL and 0.001 mg/mL (*J. regia)*, 0.418 mg/mL and 0.002 mg/mL (*E. cardamomum),* 0.827 mg/mL and 0.012 mg/mL (*C. zeylanicum)*, 0.855 mg/mL and 0.049 mg/mL (*S. officinalis)*, and 0.895 mg/mL and 0.050 mg/mL (*H. perforatum)*. Each of these plant extracts (*n* = 5, 500 µg/mL) underwent further studies to determine their Km and Vmax type of enzymatic inhibition for AChE and BuChE (Table 1; Figure S1). However, the results of these experiments were quite variable, with some extracts not following Michaelis–Menten kinetics (Figure S1).

### 3.2. BACE-1 Inhibitory Activity

**Figure 2:**
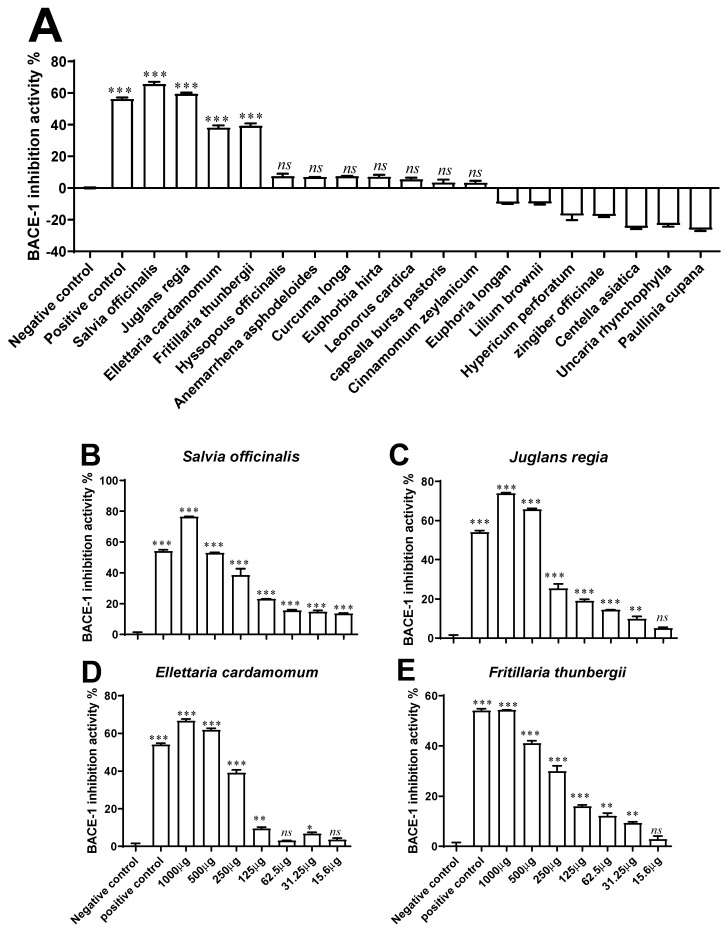
β-secretase (BACE-1) inhibitory activity of plant extracts. (**A**) Plant extract (*n* = 18) concentrations were initially screened at a concentration of 500 µg/mL; (**B**–**E**) the concentration-dependent activities (15.6–1000 µg/mL) were then determined for four selected phytoextracts, i.e., *S. officinalis, J. regia, E. cardamomum*, and *F. thunbergii.* The inhibitory activity was determined by using a standardised BACE-1 Activity Detection Kit, with KF-14 (25 µM) used as a positive control. * *p* < 0.05, ** *p* < 0.01, and *** *p* < 0.001 compared with negative control (vehicle control, i.e., 1% DMSO in ddH_2_O). *ns* (not significant).

Plant extracts (*n* = 18) were screened for BACE-1 inhibitory activity at the concentration of 500 µg/mL (Figure 2A). Among these phytoextracts, only four (*n* = 4) exhibited significant activity in a concentration-dependent manner (15.6–1000 µg/mL) based on their IC_50_ values (Figure 2B–E). These were: *S. officinalis* (IC_50_: 0.53 mg/mL), *J. regia* (IC_50_: 0.55 mg/mL), *E. cardamomum* (IC_50_: 0.60 mg/mL), and *F. thunbergii* (IC_50_: 0.81 mg/mL). The Km, Vmax, and type of enzymatic inhibition for BACE-1 of these four extracts were also assessed (Table 2; Figure S1).

### 3.3. Antioxidant Activity

**Figure 3:**
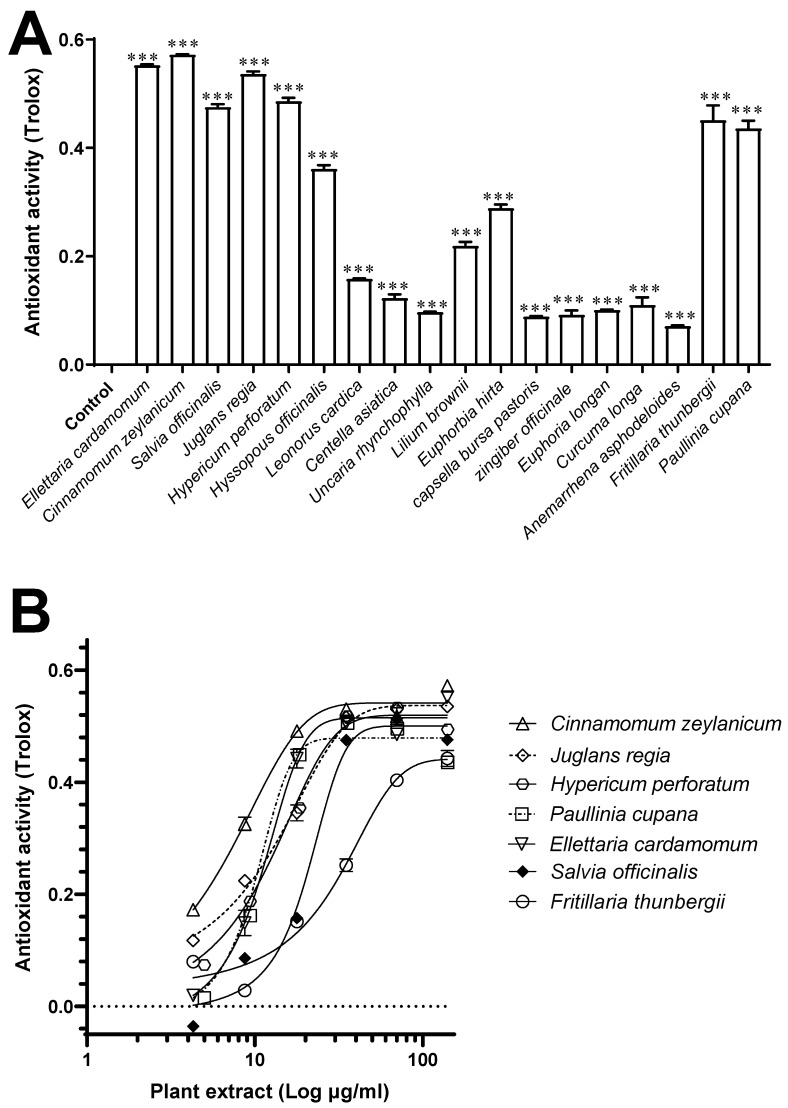
Antioxidant activity of plant extracts. (**A**) Antioxidant activity of plant extracts (*n* = 18; 140 µg/mL) was screened using a commercial ABTS antioxidant activity assay kit, with results interpolated against Trolox standard (mM); (**B**) concentration-dependent antioxidant activity (4.3–140 µg/mL) was then determined for seven selected plants (in order of greatest activity): *C. zeylanicum*, *J. regia*, *H. perforatum*, *P. cupana, E. cardamomum*, *S. officinalis*, and *F. thunbergii*. Data are mean ± SEM (*n* = 2). *** *p* < 0.001 compared with the negative control (assay buffer).

Plants extracts (*n* = 18, 140 µg/mL) were screened for their antioxidant activity. All exhibited some degree (*p* < 0.001) of antioxidant activity (Figure 3A), but the seven plant extracts (*n* = 7) that elicited the greatest antioxidant activity in a concentration-dependent manner (4.3–140 µg/mL) based on their IC_50_ values were: *C. zeylanicum*, *J. regia*, *H. perforatum*, *P. cupana, E. cardamomum*, *S. officinalis*, and *F. thunbergii*. Indeed, at a concentration of 140 μg/mL, they exerted an activity (expressed as Trolox equivalents) of 0.57 ± 0.01 mM, 0.55 ± 0.001 mM, 0.53 ± 0.003 mM, 0.48 ± 0.004 mM, 0.47 ± 0.004 mM, 0.45 ± 0.01 mM, and 0.43 ± 0.01 mM, respectively (Figure 3B).

### 3.4. Effects on Chemotaxis in G. pallida

**Figure 4:**
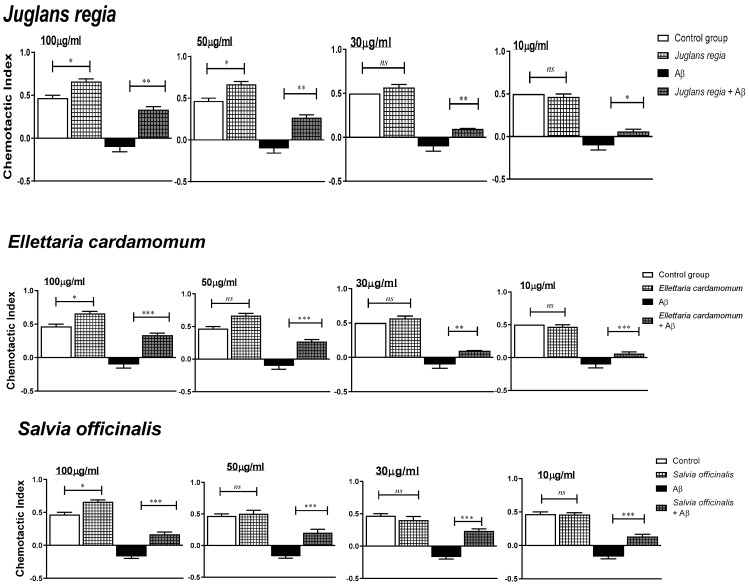

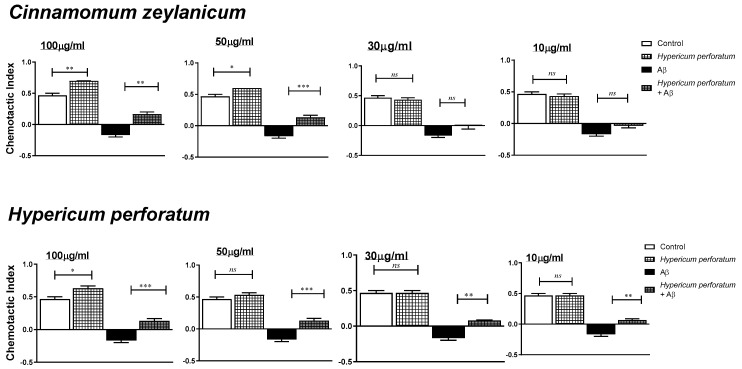
Concentration-dependent effects of plant extracts on chemotactic index (CI) in *Globodera pallida*. *G. pallida* were pre-incubated (24 h) with either: vehicle control (1% DMSO in ddH_2_O), Aβ (1-42) at 100 μM, plant extract (10–100 μg/mL) alone, or in combination with Aβ (1-42). Data are mean ± SEM (*n* = 3) with approximately 100 *G. pallida* organisms per observation. * *p* < 0.05, ** *p* < 0.01, and *** *p* < 0.001 (significant) and *ns* (not significant) for the comparisons are indicated.

The concentration-dependent effects of the selected medicinal plant extracts (*n* = 5, 10–100 µg/mL) on the chemotaxis index (CI), both in the presence and absence of Aβ (100 μM), are shown in Figure 4.

No extracts adversely affected CI at the highest concentration of 100 µg/mL, and all the plant extracts enhanced it. All the five selected plant extracts (i.e., *J. regia*, *E. cardamomum*, *C. zeylanicum*, *S. officinalis*, and *H. perforatum*) at all tested concentrations significantly (*p* < 0.05) ameliorated chemotaxis of untreated *G. pallida* (vehicle control) and achieved almost complete restoration to normal after exposure of the nematodes to Aβ (1-42)-induced impairments. The responses behaved in a dose-dependent manner.

Thereby, at the optimal plant extract concentration of 100 µg/mL, *C. Zeylanicum* enhanced effectively (*p* < 0.05) the chemotaxis of *G. pallida* (control group) by 48.93%; all three *J. regia*, *E. cardamomum*, and *S. officinalis*, by 40.42%; and to a lesser extent, H. perforatum, by 34.04%. Importantly, *G. pallida* exposed to Aβ (1-42)-induced chemotaxis impairments (−121.28% to −136.17%) were reverted significantly (*p* < 0.01) by *J. regia* (increased of 191.48%, which corresponds to a restoration of 70.20% compared to control) and *E. cardamomum* (increased of 193.58%, restoration of 63.80%), followed to a lesser extent by *S. officinalis* (increased of 172.34%, restoration of 36.17%)*, C. Zeylanicum* (increased of 172.34%, restoration of 36.17%), and *H. perforatum* (increased of 163.83%, restoration of 27.66%).

### 3.5. Phytoprotective Effects against Aβ (1-42)-Induced Impairments in G. pallida

**Figure 5:**
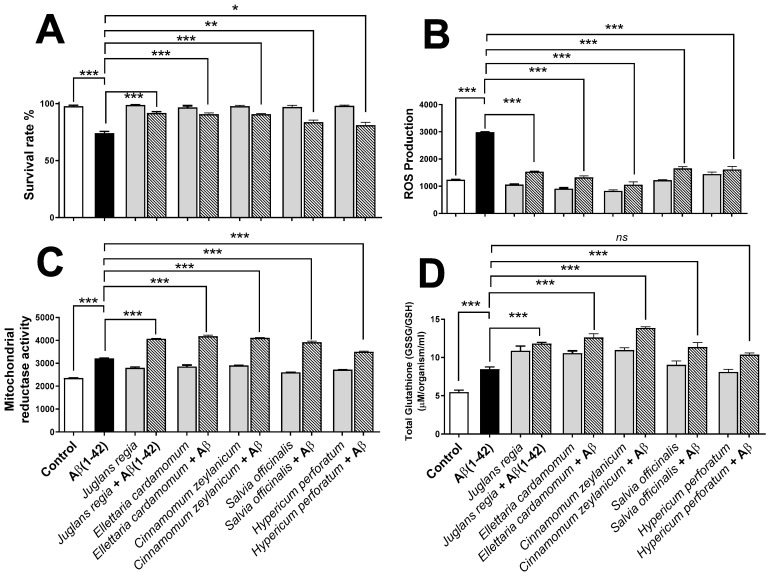
Evaluating the ability of plant extracts to ameliorate the effects of amyloid beta Aβ (1-42) in *Globodera pallida.* (**A**) Percentage survival of *G. pallida* was determined by visual counting of living/dead organisms following exposure to Aβ (1-42) alone, plant extracts, or plant extracts in combination with Aβ (1-42). (**B**) ROS production was monitored using the DCFH-DA assay, and the fluorescence (Ex: 495 nm/Em: 529 nm) was measured every 30 min for 24 h; the results are reported as areas under the curve (AUC). (**C**) For mitochondrial reductase activity, *G. pallida* were incubated with Alamar Blue, and the fluorescence (Ex: 530 nm/Em: 590 nm) was measured every 30 min for 24 h. The results are reported as AUC. (**D**) Total glutathione is the ratio of oxidized glutathione (GSSG) to reduced glutathione (GSH), which is a measure of cellular oxidative stress. It was determined by employing OxiSelect^™^ Total Glutathione assay in the presence of nicotinamide adenine dinucleotide phosphate (NADPH) at 405 nm. The total glutathione content in unknown samples is determined by comparison with the predetermined glutathione standard curve. In each type of experiment, the nematodes were exposed to Aβ (1-42) alone (100 µM), plant extracts (*n* = 5; 100 µg/mL), or plant extracts (*n* = 5; 100 µg/mL) in combination with 100 µM Aβ (1-42). Control represents untreated cells (vehicle control); heat-killed *G. pallida* (not shown) were also performed as controls. Approximately 50 *G. pallida* J2 organisms per replicate were used in survival assays, ~100 organisms were used in mitochondrial reductase and ROS assays, and glutathione studies involved ~1000 organisms per replicate. Data are mean ± SEM (*n* = 3), * *p* < 0.05, ** *p* < 0.01, *** *p* < 0.001 (significance), and *ns* (not significant) for the statistical comparisons indicated (one-way analysis of variance).

The ability of the selected plant extracts (*n* = 5, 100 µg/mL) to exert protective effects against Aβ (1-42)-induced toxicity was investigated in *G. pallida*. The effects of these plant extracts (i.e., *J. regia*, *E. cardamomum*, *C. zeylanicum*, *S. officinalis*, and *H. perforatum*) were evaluated on cell survival rate and oxidative stress indexes (i.e., total ROS production, mitochondrial reductase activity level, and oxidised/reduced glutathione ratio)*,* both in the presence and absence of 100 µM Aβ (1-42) peptide (Figure 5). Untreated cells (in medium or buffer only) were used as vehicle controls.

When incubated alone, no plant extracts did affect the survival of the nematodes (Figure 5A). Contrastingly, incubation with Aβ (1-42) significantly reduced (*p* < 0.001) the survival the nematode (Figure 5A). Interestingly, all five plant extracts (at the optimal concentration of 100 μg/mL) significantly improved (*p* < 0.05) the percentage survival rate of *G. pallida* pre-treated with Aβ (1-42). Indeed, *J. regia* increased the survival of *G. pallida* pre-treated with the peptide by 17%, *E. cardamomum* by 16%, *C. zeylanicum* by 16%, *S. officinalis* by 9%, and *H. perforatum* by 7%.

Moreover, incubation of the plant extracts alone did not affect significantly (*p* > 0.05) the ROS production in *G. pallida*, but Aβ (1-42) significantly increased (*p* < 0.001) the ROS production (Figure 5B). Importantly, all five plant extracts significantly (*p* < 0.001) reduced to almost normal the Aβ (1-42)-induced ROS production in *G. pallida.* Thereby, *C. zeylanicum* reduced ROS production by 64%, *E. cardamomum* by 55%, *J. regia* by 49%, *S. officinalis* by 40%, and *H. perforatum* by 38%.

Two common cellular protective responses were measured for plant extracts in the presence/absence of Aβ (1-42): the mitochondrial reductase activity (Figure 5C) and the GSSG/GSH ratio which reflects the total glutathione (Figure 5D). Plant extracts alone significantly increased (*p* > 0.05) both measurements, whereas Aβ (1-42) increased (*p* < 0.001) the levels of these parameters. Most important, all plant extracts (with the exception of *H. perforatum* for total glutathione, *p* > 0.05) displayed better protective responses (*p* < 0.001) in Aβ (1-42) pre-treated nematodes. Indeed, mitochondrial reductase activity was increased by *E. cardamomum* (30.33%), *C. zeylanicum* (28.14%), *J. regia* (26.77%), *S. officinalis* (22.19%), and *H. perforatum* (9.16%). Similarly, the GSSG/GSH ratio was increased (*p* < 0.01) similarly by *E. cardamomum* (66%) and *C. zeylanicum* (66%), followed by *J. regia* (46%), *S. officinalis* (27%), and *H. perforatum* (18%).

## 4. Discussion

This study undertook in vitro screening of 18 medicinal plant extracts traditionally used in the treatment of neurological conditions within ethnomedicinal systems. The phytoextracts were screened for their ability to enhance the antioxidant activity and survival rate and inhibit three enzymatic targets (i.e., AChE, BuChE, and BACE-1) in *G. pallida* (pre-treated or not with Aβ (1-42) peptide) related to the progression and development of AD. To the best of our knowledge, this is the first time that this nematode model has been employed in drug discovery studies [11,12]. Several plant candidates with promising activities were identified by in vitro testing, necessitating further in vivo investigations using *G. pallida*. Indeed, five potent extracts acted strongly against the cholinergic enzymes AChE and BuChE; four potent extracts acted against BACE-1; and seven plants elicited extraordinarily strong antioxidant activities. Extracts obtained from *J. regia, E. cardamomum, C. zeylanicum, S. officinalis,* and *H. perforatum* exerted the most potent and multi-faceted activities to protect against Aβ-induced impairment in *G. pallida* (Table S4). In addition to their greatest AchE and BuChE inhibitory activities, the effects of *J. regia* and *E. cardamomum* extracts in restoring the organism’s chemosensing and improving survival were extremely encouraging.

As a species, *J. regia* L., commonly known as walnut, as previously been reported to possess neuroprotective effects in in vitro [17,18] and in vivo studies [19,20,21,22,23]. However, the major distinction between the present study and such studies is that these previous studies almost exclusively focused on the edible fruit of this plant (walnut), and not the leaves. Indeed, the studies arose from epidemiological evidence associating diets rich in walnuts with lower incidence of neurodegenerative disease. However, leaves of *J. regia* have been used in traditional medicine for thousands of years in the treatment of many conditions. Flower and leaf aqueous ‘tea infusions’ of *J. regia* were reported to inhibit AChE by up to 45% [18], an action that may be attributed to gallic acid, ellagic acid, and other related compounds [24,25]. The present study, using a ethanol:water extraction, shows that much greater levels of activity can be achieved, reaching up to 73% inhibition against AChE and 90% inhibition against BuChE. Such levels of activity can be compared very favourably with the purified galantamine. Other studies investigating AChE inhibitory activity of *J. regia* [17,26] have had mixed levels of success, indicating that the measurement methods and the plant part extracts are also important. There has been a pre-ponderance of studies investigating the anti-amyloidogenic activity of ‘walnut extracts’; however, these were compositionally very different from leaf extracts [27,28]. Herein, we show for the first time that *J. regia* leaves possess compounds not only with BACE-1 inhibitory activity but also protective action against Aβ-induced damage (as demonstrated by several different assessments). Along with the other four extracts, *G. pallida*’s chemosensing was enhanced by *J. regia* in the absence of Aβ, but comparatively to other selected phytoextracts, *J. regia* extracts achieved this activity at much lower concentrations. Furthermore, in the presence of Aβ, leaves extract of *J. regia* (i) significantly improved the nematode’s chemosensing at all concentrations tested, restoring up to 70% of lost chemosensory capability; (ii) improved the survival rate of the nematode to the greatest extent, which was restored from a level of 74% to 92%, almost reaching the levels of control incubations; (iii) led to a strong reduction in ROS production, which, again, was almost completely normalised; and (iv) significantly augmented oxidative stress defence responses. The detailed mechanisms underlying these protective effects remain to be unpicked. The main source of ROS biologically is NADPH oxidase, and oligomeric Aβ (1–42) induces ROS production by activating this enzyme [29]. Therefore, we can hypothesize that NADPH oxidase activity was impacted by compounds from *J. regia* and other shortlisted plants that were shown to possess a strong antioxidant activity, which is known to counteract Aβ toxicity [30,31]. What is clear is that both the glutathione and mitochondrial reductase systems were augmented when *G. pallida* was exposed to leaf extract of *J. regia* and improved the principal defensive capabilities of the organism against Aβ. Further investigations could examine whether heat shock proteins (HSPs), transcription factors, and/or other antioxidant enzymes in *G. pallida* are altered, since these molecules have been investigated in other model systems [30,32].

The second very promising ethanolic extract was *E. cardamomum* (L.), commonly known as cardamom, which inhibited AChE and BuChE to a similar extent to galantamine, and to a greater magnitude compared to other reports [33]. *E. cardamomum* is cultivated commercially in India and Sri Lanka. Its traditional use is in the treatment of gastrointestinal disorders, but it is also used as an anti-depressant which, over a prolonged period, can enhance cognitive function [33,34]. In vivo studies suggest that alcoholic extracts of *E. cardamomum* have anxiolytic, anti-depressant, and cognition-enhancing properties, possibly due to its flavonoid content [34,35]. The anxiolytic effects of *E. cardamomum* have been particularly attributed to the high flavonoid content and quercetin levels [34]. In many respects, the protective actions of *E. cardamomum* against Aβ were like that of *J. regia,* with a remarkable ability to restoring the chemosensing of *G. pallida,* and so this plant extract should also be prioritised for further investigations.

Although not as potent in restoring chemosensing abilities, some of other plant extracts tested had interesting properties. We observed that the ethanolic flowers extracts of *H. perforatum* (L.), known as St. John’s Wort, and *S. officinalis* (L.), also called sage, had greater propensity to inhibit BuChE than AChE, albeit with a degree of selectivity for BuChE, suggesting that there might have compounds that should be purified from these two extracts. This appears to be a new observation, but previous studies using methanolic extracts of *H. perforatum* observed similar maximal levels of AChE inhibition indicating a good data reproducibility [36,37,38]. The inhibitory activity of *S. officinalis* against AChE has been previously reported, and other studies have also reported the ability of various sage species in enhancing cognitive function [39,40,41,42]. Traditionally, *S. officinalis* has a very longstanding reputation for improving memory and cognitive function. Many bioactive phytochemicals have been isolated from *S. officinalis*, mainly phenolic diterpenes, which possess diverse biological activities [39]. It is thought that the ability of *S.* *officinalis* extracts to improve memory and cognitive functions relates to its inhibition of AChE [40,43,44], and possibly its neuroprotective effect against Aβ-induced toxicity [45]. Isorosmanol and 7-methoxyrosmanol isolated from *S. officinalis* have been previously reported to inhibit AChE by 50–65% at concentrations of 500 µM [39,46]. Cytoprotective effects of sage against Aβ in neuronal cells have been reported [45], and oral administration of its essential oil to patients improves cognition and memory with no adverse effects [47,48]. Interestingly, we also found that, from all the extracts tested herein, *S. officinalis* achieved the highest BACE-1 inhibitory activity (up to 76%). In vitro and in vivo studies have reported that salvia species contain a large array of active compounds that may enhance cognitive activity, protect against neurodegenerative diseases, and may be beneficial in preventing the onset of age-related dementia [40,49]. Compounds present in salvia plants (which may offer either cellular protection against Aβ or inhibition of BACE-1) include rosmarinic acid, salvianolic acid, carnosic acid, quercetin, and tanshinone [45,50,51,52,53,54,55]. It is also interesting that *S. officinalis* protects mice from Aβ-induced neurotoxicity by inhibiting increases of tumour necrosis factor-α (TNF-α) levels, because this suggests that other mechanisms (such as re-balancing cytokines and neurotrophins levels) are important in preventing learning and memory deficits [56].

The ethanolic extract with most potent antioxidant activity was from the bark of *C. zeylanicum* (Linn.), known as cinnamon, which has been used as a spice and traditional medicine for many centuries [57]. Cinnamon significantly inhibits tau protein aggregation, promotes the disassembly of recombinant tau filaments, and substantially alters the morphology of paired-helical filaments isolated from AD brain [58]. Furthermore, oral administration of cinnamon reduces Aβ oligomerization and improves the cognitive behaviour of mouse models of AD pathology [58]. *C. zeylanicum* can improve the activity of antioxidants enzymes in vivo, lowering malondialdehyde (MDA) levels in humans [59,60,61,62]. Evidence suggests that the bioactivity of cinnamon is largely ascribed to phenolic compounds, including cinnamic acid, cinnamate, cinnamaldehyde, and proanthocyanidins [63,64].

In short, this study identified several promising plant extracts with pleiotropic functions representing a huge therapeutic potential in AD. Moreover, the recently described *G. pallida* model system was employed for the first time in drug screening, demonstrating its utility in drug discovery research. The strengths of this investigation include the (i) selection of plants based on ethnomedicinal use, (ii) stepwise use of in vitro and in vivo approaches, (iii) detailed concentration-dependent testing, and (iv) probing of enzyme inhibitory mechanisms. One limitation was that the broad screening approach made it impractical to optimize, standardize, and determine the reproducibility of the extraction procedure. Nonetheless, this study has brought forward a range of new information; for instance, BACE-1 inhibition has not previously been assessed in many of the plants herein reported, except for *C. zeylanicum* and *C. longa* [65,66].

Future studies should characterize the biochemical composition of *J. regia* and *E. cardamomum* extracts using techniques such as HPLC and perform pre-clinical and clinical testing to evaluate them as alternative or adjunct therapies. Inexpensive and effective treatments based on medicinal plants remain a viable way of reducing the global burden of AD in developing countries. Furthermore, it will be necessary to (i) extract, fractionate, and isolate candidate compounds before subjecting them to repeated rounds of screening in *G. pallida* and (ii) discover novel compounds/chemical scaffolds with protective effects against Aβ-induced toxicity.

## Figures and Tables

**Table 1:** AChE and BuChE inhibitory properties of selected plant extracts.

		Controls		Screened Plant Extract (*n* = 5) ± Std. Error				
	Kinetics Properties	NC	PC	*J. r*	*E. c*	*C. z*	*S. o*	*H. p*
AChE	Vmax (mM/min)	0.008651 ± 0.0005743	0.00869 ± 0.0004418	0.00849 ± 0.0003554	0.003548 ± 0.0002209	0.004507 ± 0.0004567	0.00434 ± 0.0004683	0.00469 ± 0.0004768
	Km (mM)	0.003523 ± 0.001149	0.01441 ± 0.002464	0.01092 ± 0.001659	0.001344 ± 0.0006181	0.01951 ± 0.006144	0.01644 ± 0.005772	0.00246 ± 0.001398
	Proposed Inhibitory type	N/A	competitive	competitive	mixed	mixed	mixed	uncompetitive
BuChE	Vmax (mM/min)	0.00753 ± 0.0007588	0.00565 ± 0.001249	0.00391 ± 0.0004516	0.002214 ± 0.0002146	0.005219 ± 0.0008111	0.00372 ± 0.0003888	0.00383 ± 0.0006241
	Km (mM)	0.09534 ± 0.03577	0.43380 ± 0.2367	0.0938 ± 0.04096	0.02058 ± 0.01200	0.3486 ± 0.1430	0.14000 ± 0.04964	0.09475 ± 0.05747
	Proposed Inhibitory type	N/A	mixed	uncompetitive	mixed	mixed	mixed	uncompetitive

Vmax, Km, and the proposed type of acetylcholinesterase (AChE) and butyrylcholinesterase (BuChE) inhibitory activities are mentioned. Kinetic studies involved fixed concentrations of the Galantamine inhibitor (18 µM for AChE or 2 µM for BuChE) or screened phytoextracts (*n* = 5; 500 µg/mL) and variable substrate concentrations (i.e., acetylthiocholine iodide (ATCI): 0.0023–0.15 mM for AchE inhibition assay or butyrilthiocholine chloride (BTCC): 0.023–1.5 mM for BuChE inhibition assay). Km and Vmax parameters were calculated by Michaelis–Menten curve fitting. N/A = not applicable. NC = negative control (no inhibitor); PC = positive control (Galantamine). *J. r*: *Juglan regia; E. c*: *Ellettaria cardamomum; C. z*: *Cinnamomum zeylanicum*; *S. o*: *Salvia officinalis*; *H. p*: *Hypericum perforatum*.

**Table 2:** BACE-1 inhibitory property of selected plant extracts (*n* = 4).

		Controls		Screened Plant Extract (*n* = 4)			
	Kinetics Properties	NC	PC	*J. r*	*E. c*	*F. t*	*S. o*
BACE-1	Vmax (mM/min)	361.9	361.7	276.5	351.0	361.3	120.3
	Km (mM)	2.881	6.062	6.893	4.627	4.82	0.9230
	Proposed Inhibitory type	N/A	competitive	mixed	mixed	competitive	uncompetitive
	IC50 (mg/mL)	N/A	25 µM	0.55	0.60	0.81	0.53

Vmax, Km, the proposed type of the β-secretase inhibitory activity, and IC50 values are mentioned. Kinetic studies involved fixed concentrations of the KF-14 inhibitor (25 µM) or phytoextract (*n* = 4; 500 µg/mL) and variable substrate concentrations (0.156–10 mM). Km and Vmax parameters were calculated by Michaelis–Menten curve fitting. N/A = not applicable. NC = negative control (no inhibitor); PC = positive control (KF-14). *J. r*: *Juglan regia*; *E. c*: *Ellettaria cardamomum*; *F. t*: *Cinnamomum zeylanicum*; *S. o*: *Salvia officinalis*.

## Data Availability

Data sharing upon request to N.A.A., F.M., or B.D.G.

## Supplementary Materials

The following supporting information can be downloaded at: https://www.mdpi.com/article/10.3390/antiox11101865/s1, Figure S1: Inhibitory properties of *Juglans regia* extract compared with known inhibitors; Table S1: Extracts of ethnomedicinal plants (*n* = 18) used in this study. The ethanolic extract yield for each plant’s part is specified; Table S2: % acetylcholinesterase inhibitory activity of ethanolic plant extracts (*n* = 18); Table S3: % butyrylcholinesterase inhibitory activity of plant extracts (*n* = 18); Table S4: Summary of in vitro and in vivo (in *G. pallida* ± pre-treated with Aβ (1–42) peptide) activities exerted by the screened ethanolic phytoextracts (*n* = 5).
