# Supplementary material for: Ethnomedicinal Plants with Protective Effects against Beta-Amyloid Peptide (Aβ)1-42 Indicate Therapeutic Potential in a New In Vivo Model of Alzheimer’s Disease
Source: Antioxidants (Basel). 2022 Sep 21;11(10):1865. doi: 10.3390/antiox11101865 (PMC9598277; doi:10.3390/antiox11101865)
Supplement: Supplementary file 1 [file antioxidants-11-01865-s001.zip › antioxidants-1808488-supplementary.pdf]

antioxidants

MDPI

Supplementary Materials

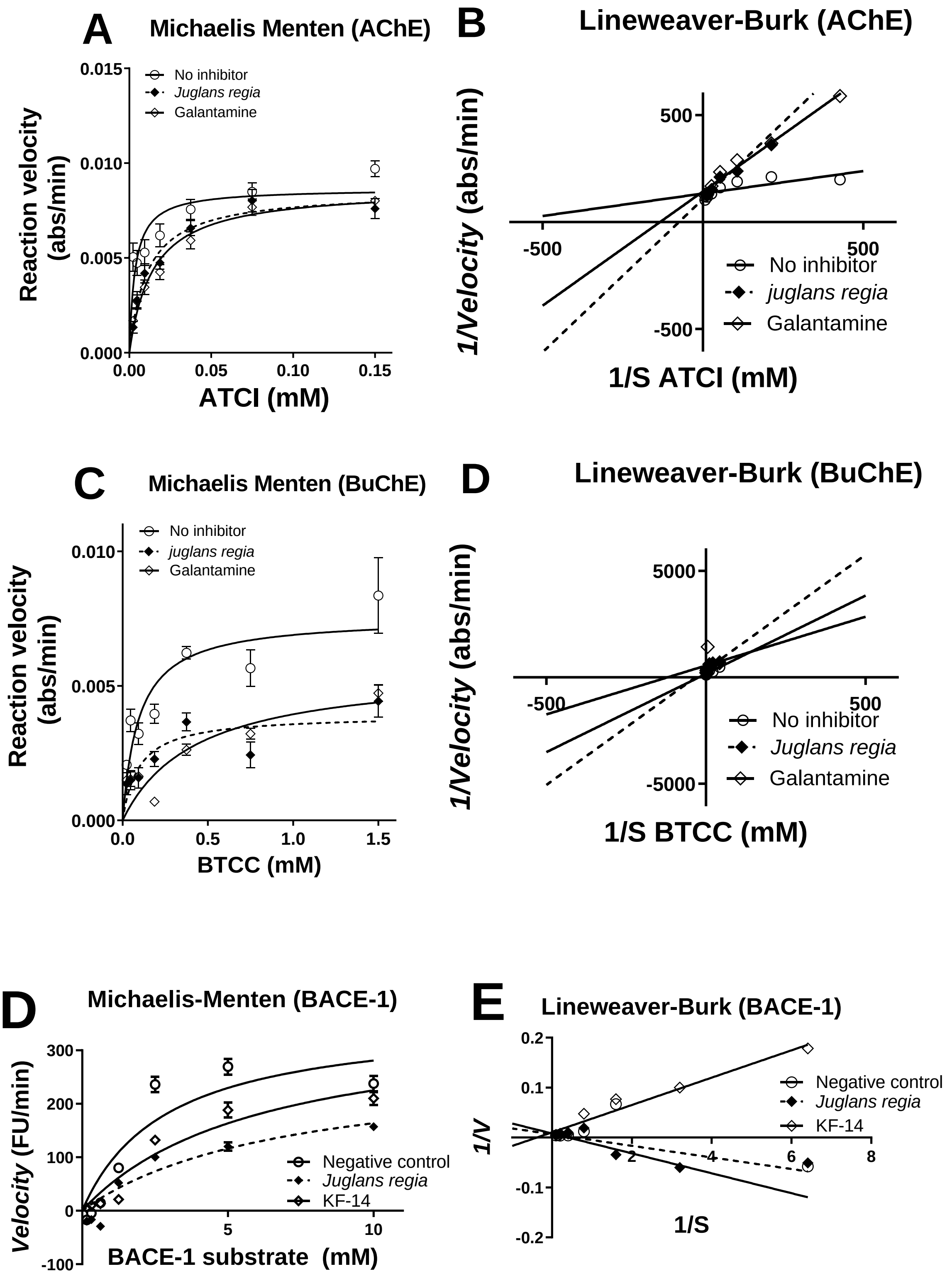

Figure S1. Inhibitory properties of *Juglans regia* extract compared with known inhibitors. (**A**) and (**C**) show the Michaelis–Menten curves and (**B**) and (**D**) the Lineweaver–Burk plots for acetylcholinesterase (AChE) and butyrylcholinesterase (BuChE) inhibitory properties of *J. regia.* Substrate

concentrations were varied in the presence of a fixed amount of *J. regia* extract or galantamine. It was determined that both *J. regia* and galantamine are competitive inhibitors of AchE. For BuChE, *J. regia* acted in a non-competitive manner and galantamine was a 'mixed' inhibitor. (**E**) show the Michaelis–Menten curves and the Lineweaver–Burk plots for β-secretase 1 (BACE-1) inhibitory properties of *J. regia*. Substrate concentrations were varied in the presence of a fixed amount of *J. regia* extract or KF-14. It was determined that *J. regia* extract had a 'mixed' mode of action whereas KF-14 was a competitive inhibitor for BACE-1.

Table S1. Extracts of ethnomedicinal plants (n = 18) used in this study. The ethanolic extract yield for each plant's part is specified.

#	Botanical Name/ Common Name	Plant Family	Region of Origin	Traditional Use/Treatment/Benefits in Neurology	Part(s) Used	Extract Yield (%)*
1	*Anemarrhena asphodeloides*/ Zhi mu	Asparagaceae	China	Neurodegenerative diseases [67].	Rhizome	26.32
2	*Capsella bursa-pastoris*/ Shepherd's Purse	Brassicaceae	Europe	Memory [68].	Herb	5.24
3	*Centella asiatica*/ Gotu Kola	Umbelifers	Asia	Anxiety, cognition and emotional disorders [69].	Leaf	9.06
4	*Cinnamomum zeylanicum*/ Cinnamon	Lauraceae	Sri Lanka	Nervous stress, 'nervine tonic' [70].	Bark	3.11
5	*Curcuma longa*/ Tumeric	Zinziberaceae	Southern Asia	Depression [71].	Rhizome	3.62
6	*Ellettaria cardamomum*/ Cardamom	Zingiberaceae	South India	Anxiety [72].	Seed	8.10
7	*Euphorbia hirta*/ Snakeweed	Euphorbiaceae	India	Anxiety [73].	Leaf	7.20
8	*Euphoria longan*/ Longan	Sapindaceae	Southern China	Learning and memory [74].	Fruit	22.19
9	*Fritillaria thunbergii*/ Zhe Bei Mu	Liliaceae	China	Epilepsy, Neuroprotection [75,76].	Bulb	1.83
10	*Hypericum perforatum*/ Saint John's wort	Hypericaceae	Europe, Asia	Depression and anxiety [77].	Flower	8.12
11	*Hyssopus officinalis*/ Hyssop	Lamiaceae	Europe	Nervous disorders [78].	Flower, young leaf	2.53
12	*Juglans regia*/ Walnut	Junlandaceae	Central Asia	Neuroprotection, memory, and learning [79].	Leaf	8.02
13	*Leonurus cardiaca*/ Motherwort	Lamiaceae	Europe, Asia	Sleep disorders and anxiety [80].	Aerial	5.23
14	*Lilium brownie*/ Lily bulb	Lamiaceae	Europe	Anxiety and apprehension [81].	Flower, young leaf	6.64
15	*Paullinia cupana*/ Saint John's wort	Hypericaceae	Europe, Asia	Cognition [82].	Flower	6.82
16	*Salvia officinalis*/ Sage	Lamiaceae	Mediterrane an	Memory [83].	Flower and leaf	6.62
17	*Uncaria rhynchophylla*/ Cat's Claw Walnut	Junlandaceae	Central Asia	Epilepsy [84].	Leaf	3.63
18	*Zingiber officinale*/ Ginger	Zingiberaceae	Southeast Asia	Anxiety [85].	Rhizome	1.23

*Percentage yield of extracts obtained after extraction of plant materials in 70% ethanol solution.

Table S2. % acetylcholinesterase inhibitory activity of ethanolic plant extracts (*n* = 18). NC= Negative control Sodium phosphate buffer); PC= Positive control (Galantamine 18 μM).

Plant Extract (mg/mL)	NC (%)	PC (%)	25 (%)	50 (%)	100 (%)	250 (%)	500 (%)
Anemarrhena asphodeloides	0.585578	59.333	22.56158	24.81351	10.47994	31.71881	26.19207
	−0.585578	58.93703	21.57635	21.71147	16.27305	32.73939	21.58552
Capsella bursa pastoris	0.585578	59.333	21.54258	39.20901	34.72203	40.04239	33.77391
	−0.585578	58.93703	17.03871	26.67136	32.47009	39.797	31.15833
Centella asiatica	0.585578	59.333	21.54258	39.20901	34.72203	40.04239	33.77391
	−0.585578	58.93703	17.03871	26.67136	32.47009	39.797	31.15833
Cinnamomum zeylanicum	0.585578	59.333	31.32442	33.51443	51.34412	41.20796	41.44777
	−0.585578	58.93703	26.21253	36.88107	53.59606	41.39758	44.30874
Curcuma longa	0.585578	55.333	18.9401	27.65249	24.37435	22.6312	22.33004
	−0.585578	56.93703	19.49563	26.45344	25.83741	21.92293	23.66851
Ellettaria cardamomum	0.585578	59.333	28.54046	27.66784	32.60521	48.38269	53.06174
	−0.585578	58.93703	28.70373	31.7551	37.34553	49.13557	53.39078
Euphorbia hirta	0.9834	56.4324	26.0235	25.7509	24.8744	35.549	37.524
	−0.9834	63.2416	26.5633	24.2865	23.891	35.1379	35.654
Euphorbia longan	0.9834	56.4324	23.6505	23.0358	22.0631	24.9726	23.1541
	−0.9834	63.2416	22.0096	22.806	32.4425	21.2337	24.5243
Fritillaria thunbergii	0.9834	56.4324	22.9102	25.8712	24.8343	33.3711	26.3428
	−0.9834	63.2416	26.9562	26.3789	24.7595	32.0276	27.6214
Hyssopous officinalis	0.9834	56.264	25.0722	24.9332	22.3677	29.9257	30.6606
	−0.9834	63.4099	27.3918	24.6232	21.1331	33.0929	30.3544
Hypericum perforatum	0.9834	56.4324	30.5104	31.0396	32.4666	47.3703	39.536
	−0.9834	63.2416	31.6756	33.728	30.0294	48.1043	36.226
Juglans regia	0.9834	56.264	32.3864	32.1726	27.512	73.5347	59.4795
	−0.9834	63.4099	37.4586	33.2362	28.4206	72.708	59.1165
Lilium brownie	0.9834	56.264	23.1187	22.675	24.2036	24.3482	23.2809
	−0.9834	63.4099	22.9102	26.1384	24.5724	25.8881	22.8609
Paullinia cupana	0.9834	56.4324	23.6505	23.0358	22.0631	31.6558	36.8221
	−0.9834	63.2416	22.0096	22.806	32.4425	33.1825	37.067
Uncaria rhynchophylla	0.9834	56.4324	26.0235	25.7509	24.8744	32.1239	34.3811
	−0.9834	63.2416	26.5633	24.2865	23.891	31.8395	30.4134
Zingiber officinale	0.585578	59.333	26.01617	27.84225	29.21181	30.89064	33.87429
	−0.585578	58.93703	24.80612	23.96458	25.83466	32.23468	31.61006
Leonorus cardica	0.585578	59.333	32.63297	25.55415	28.61779	36.53728	31.36189
	−0.585578	58.93703	34.24454	27.60574	22.39701	35.97959	33.68747
Salvia officinalis	0.585578	59.333	30.97537	26.92189	25.019	41.51191	37.34036
	−0.585578	58.93703	25.18227	28.08726	28.75721	40.19575	38.39998

Table S3. % butyrylcholinesterase inhibitory activity of plant extracts (*n* = 18).

Plant Extract (mg/mL)	NC (%)	PC (%)	25 (%)	50 (%)	100 (%)	250 (%)	500 (%)
Anemarrhena asphodeloides	0.9690363	74.03226	41.63071	37.93626	34.15096	59.46066	52.57104
	−0.9690363	73.12564	37.5653	38.06496	36.44485	50.74366	44.06694
Capsella bursa pastoris	0.9690363	74.03226	42.21364	40.0106	43.68234	61.21707	45.98303
	−0.9690363	73.12564	47.16481	43.24324	43.81861	61.94476	54.15595
Centella asiatica	−2.128794	74.03226	50.20666	47.33567	48.37563	64.87258	66.75694

	2.128794	73.12564	53.91316	51.7177	50.45554	66.43247	67.46793
Cinnamomum zeylanicum	−0.2820947	73.69852	65.03052	65.36903	67.5745	77.7726	86.3498
	0.2820947	75.03206	65.95374	68.68236	70.14925	78.2025	85.99456
Curcuma longa	0.9690363	74.03226	54.35759	56.01085	42.89143	57.35301	55.34421
	−0.9690363	73.12564	43.15808	51.77992	43.98471	54.50869	52.58878
Ellettaria cardamomum	−0.8939882	74.03226	49.71588	55.62996	63.80976	81.91755	84.45112
	0.8939882	73.12564	53.67549	56.09324	63.67223	81.67143	84.04575
Euphorbia hirta	−0.8939882	74.03226	49.27431	40.34167	43.2227	58.73539	58.27211
	0.8939882	73.12564	43.62807	42.62481	45.76351	59.7271	57.38898
Euphorbia longan	2.018247	73.69828	61.66252	55.33131	51.24873	52.59561	52.53225
	−2.018247	75.14515	60.34467	58.15132	56.92563	55.66418	47.12831
Fritillaria thunbergii	2.018247	73.69828	64.8696	68.62437	62.18164	66.99134	66.64059
	−2.018247	75.14515	68.80472	66.86434	63.35204	68.66409	64.7694
Hyssopous officinalis	2.018247	73.69828	57.14681	52.83384	56.35425	66.52299	69.39452
	−2.018247	75.14515	60.42761	59.96682	54.42816	65.31592	69.19178
Hypericum perforatum	−0.2820947	73.69852	58.98343	57.74222	57.28061	78.27794	63.60466
	0.2820947	75.03206	60.62471	59.93743	56.3779	79.30779	69.14397
Juglans regia	−0.8939882	72.10178	49.65435	45.10116	49.65435	89.09325	69.81848
	0.8939882	71.58782	48.42376	43.60987	48.42376	91.28996	68.53031
Lilium brownii	0.5963488	74.03226	45.75787	31.25526	44.03834	51.0116	55.70922
	−0.5963488	73.12564	46.71642	39.91146	48.80182	49.44572	54.2531
Paullinia cupana	0.6321017	74.03226	62.21762	66.26534	64.83558	63.29762	65.91337
	−0.6321017	73.12564	61.81127	67.61231	67.32636	68.72005	62.60861
Uncaria rhynchophylla	0.6321017	74.03226	49.88336	48.84491	53.67597	47.28229	47.96415
	−0.6321017	73.12564	42.79479	49.55979	48.19776	47.33424	48.46419
Zingiber officinale	0.5963488	74.03226	39.42121	35.60897	39.06267	57.82388	62.52881
	−0.5963488	73.12564	43.6798	29.83573	36.31142	60.23854	60.86782
Leonorus cardica	0.9690363	74.03226	48.74631	41.47173	39.81376	44.95491	65.85352
	−0.9690363	73.12564	49.10144	40.20744	48.24741	49.82903	66.79382
Salvia officinalis	−0.2820947	73.69852	60.91798	64.27204	74.24686	75.56901	84.61784
	0.2820947	75.03206	60.0616	63.46725	70.0616	75.78443	83.69929

NC= Negative control (Sodium phosphate buffer); PC= Positive control (Galantamine 2 μM).

Table S4. Summary of *in vitro* and *in vivo* (in *G. pallida* ± pretreated with Aβ(1–42) peptide) activities exerted by the screened ethanolic phytoextracts (n = 5). The relative effects are specified from (-) : insufficient or not screened/determined effect to $(+)_4$, or $(+)_5$, or $(+)_7$: highest relative effect for a given parameter.

	In Vitro Screening (from n = 18 plant extracts)					Potential In Vivo AD Protecting Effects			
**Plant extract**	**AchE (n = 5)**	**BuChE (n = 5)**	**BACE1 (n = 4)**	**Antioxidant (n = 7)**	**CI (n = 5)**	**Survival (n = 5)**	**ROS (n = 5)**	**MR (n = 5)**	**GSSH/GSH (n = 5)**
J. r	+++++	+++++	+++	++++++	+++++	+++++	+++	+++	+++
E. c	++++	++++	++	+++	++++	++++	++++	+++++	+++++
C. z	+++	+++	-	+++++++	+++	+++	+++++	++++	++++
S. o	++	++	++++	++	++	++	++	++	++
H. p	+	+	-	+++++	+	+	+	+	+
F. t	-	-	+	+	-	-	-	-	-
P. c	-	-	-	++++	-	-	-	-	-

J. r : Juglan regia; E. c : Ellettaria cardamomum; C.z : Cinnamomum zeylanicum; S. o : Salvia officinalis; H. p : H. perforatum; F. t : F. thunbergii; P. c : P. cupana; AchE : acetylcholinesterase; BuChE : butylcholinesterase; BACE1 : beta-secretase

1; CI : Chemotaxis index; ROS : Reactive oxygen species; MR : Mitochondrial reductase; GSSH/GSH ratio : oxidized glutathione/reduced glutathione ration (= total glutathione).
